# A nomogram based on endothelial function and conventional risk factors predicts coronary artery disease in hypertensives

**DOI:** 10.1186/s12872-023-03235-6

**Published:** 2023-04-28

**Authors:** Xiao-Dong Huang, Ji-Yan Lin, Xian-Wei Huang, Ting-Ting Zhou, Liang-Di Xie

**Affiliations:** 1 Department of Emergency, The First Affiliated Hospital of Xiamen University, Xiamen, 361003 China; 2 Fujian Hypertension Research Institute, The First Affiliated Hospital of Fujian Medical University, Fuzhou, 350005 China; 3 Xiamen Key Laboratory for Clinical Efficacy and Evidence-Based Research of Traditional Chinese Medicine, The First Affiliated Hospital of Xiamen University, Xiamen, 361003 China; 4 Department of Cardiovascular Medicine, Xiamen Haicang Hospital, Xiamen, 361026 China; 5 Department of Geriatrics, The First Affiliated Hospital of Fujian Medical University, Fuzhou, 350005 China

**Keywords:** Essential hypertension, Coronary artery disease, Flow-mediated dilation, Endothelial function, Nomogram

## Abstract

**Background:**

There is currently a lack of a precise, concise, and practical clinical prediction model for predicting coronary artery disease (CAD) in patients with essential hypertension (EH). This study aimed to construct a nomogram to predict CAD in patients with EH based on flow-mediated dilation (FMD) of brachial artery and traditional risk factors.

**Methods:**

Clinical data of 1752 patients with EH were retrospectively collected. High-resolution vascular ultrasound was used to detect FMD in all patients at the Fujian Hypertension Research Institute, China. Patients were divided into two groups, i.e. training group (n = 1204, from August 2000 to December 2013) and validation group (n = 548, from January 2014 to May 2016) according to the time of enrollment. Independent predictors of CAD were analyzed by multivariable logistic regression in the training group, and a nomogram was constructed accordingly. Finally, we evaluated the discrimination, calibration, and clinical applicability of the model using the area under curve (AUC) of receiver operating characteristic analysis, calibration curve combined with Hosmer-Lemeshow test, and decision curve, respectively.

**Results:**

There were 263 (21.8%) cases of EH combined with CAD in the training group. Multivariate logistic regression showed that FMD, age, duration of EH, waist circumference, and diabetes mellitus were independent influencing factors for CAD in EH patients. Smoking which was close to statistical significance (*P* = 0.062) was also included in the regression model to increase the accuracy. Ultimately, the nomogram for predicting CAD in EH patients was constructed according to above predictors after proper transformation. The AUC values of the training group and the validation group were 0.799 (95%*CI* 0.770–0.829) and 0.836 (95%*CI* 0.787–0.886), respectively. Calibration curve and Hosmer-Lemeshow test showed that the model had good calibration (training group: *χ*^*2*^ = 0.55, *P =* 0.759; validation group: *χ*^*2*^ = 1.62, *P =* 0.446). The decision curve also verified the clinical applicability of the nomogram.

**Conclusion:**

The nomogram based on FMD and traditional risk factors (age, duration of EH disease, smoking, waist circumference and diabetes mellitus) can predict CAD high-risk group among patients with EH.

## Background

Essential hypertension (EH) is a chronic disease caused by multiple factors, affecting the function and structure of the heart and blood vessels [1]. Epidemiological surveys have shown that the number of patients with EH in China has reached 245 million, with north-south and urban-rural differences [2]. Coronary artery disease (CAD) is a common complication of EH and increases the risk of early death and deterioration of cardiac function in patients with EH [3, 4]. Therefore, risk assessment of CAD and primary prevention for high-risk groups can effectively curb the occurrence of atherosclerotic cardiovascular disease (ASCVD) [5]. Studies have shown that endothelial dysfunction is not only the initial link of EH vascular damage, but also the pathophysiological basis of CAD and a predictor of adverse outcome of CAD [6–9]. Hence endothelial dysfunction can connect EH with CAD [10–12].

In recent years, China and Western countries have developed many risk prediction models for CAD [5, 13, 14]. However, these models were built based only on traditional risk factors including age, course of EH, smoking, obesity and diabetes mellitus for the whole population and did not incorporate endothelial function indicators. Given the limitations of existing models, it is of great clinical necessity and value to build a more accurate, concise and practical CAD prediction model for EH population based on the combination of endothelial function and traditional risk factors. Therefore, after retrospective analysis of the clinical data of EH patients, this study established a nomogram model to predict CAD based on endothelial function indicators and risk factors included in the China-PAR study [5], and validated and evaluated the model. This model can provide a clinical reference for medical workers to identify CAD high-risk groups early and formulate corresponding prevention and treatment measures.

## Methods

### Study population

**Fig. 1:**
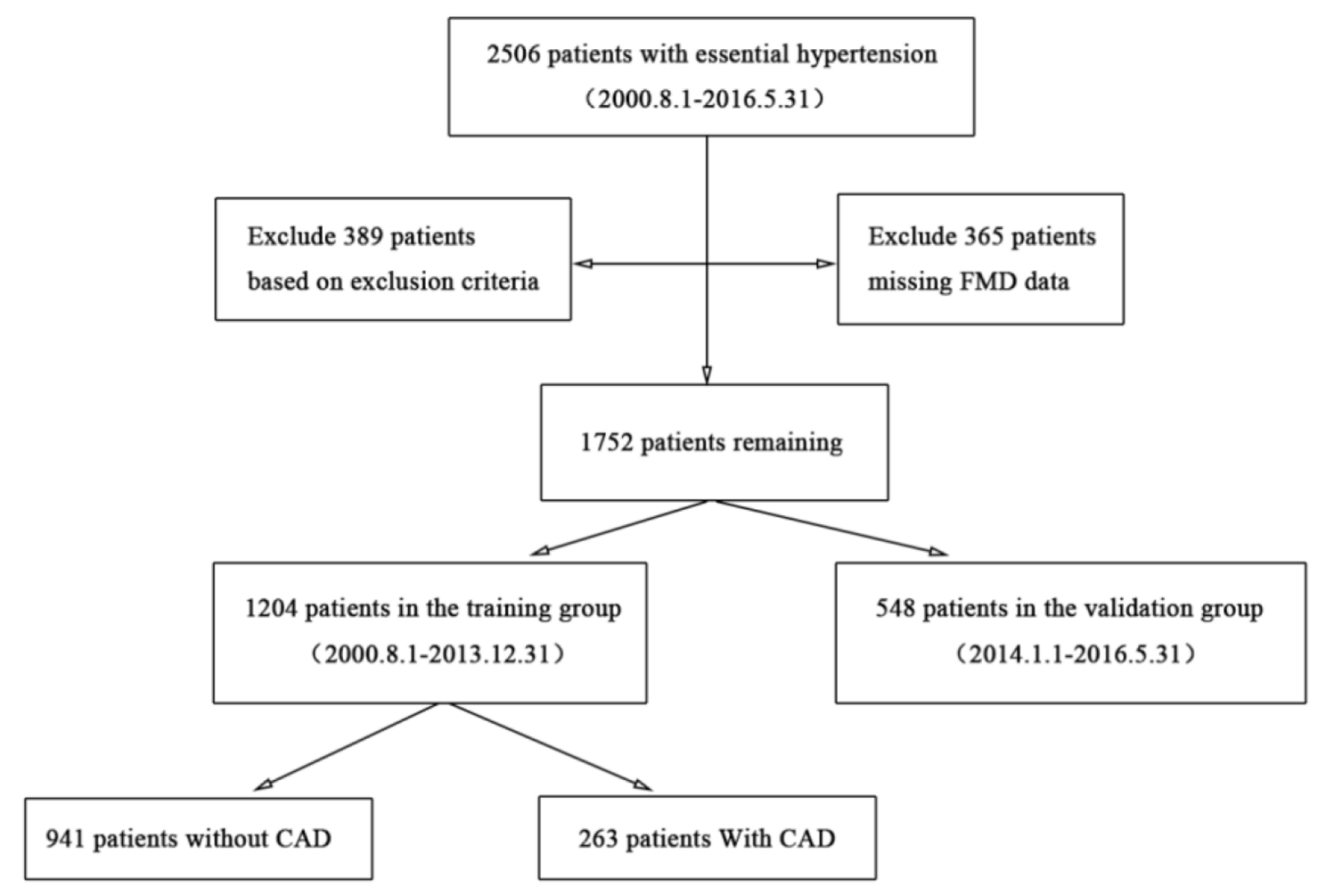
Flow chart of participant selection in this study. Abbreviations: CAD, coronary artery disease; FMD, flow-mediated dilation

2506 EH patients admitted to the First Affiliated Hospital of Fujian Medical University from August 2000 to May 2016 were included and analyzed. This study was approved by the Chinese Clinical Trial Registration Register (registered number: ChiCTR2000039448 (28/10/2020), URL:http://www.chictr.org.cn/index.aspx) and the Ethics Committee of the hospital (Affiliated I [2020] No.306) on September 15th, 2020. The included EH patients were required to meet the diagnostic criteria in the *2018 Chinese Guidelines for Prevention and Treatment of Hypertension* [15], but the patients with following conditions were excluded: secondary hypertension (n = 31), congenital heart disease (n = 26), valvular heart disease (n = 24), heart failure (n = 85), severe arrhythmias (n = 56), stroke (n = 38), peripheral vascular disease (n = 34), severe hepatorenal insufficiency (n = 28), hyperthyroidism or hypothyroidism (n = 11), hematological diseases (n = 17), rheumatic diseases (n = 5), severe infection within one month (n = 9), long-term use of corticosteroids (n = 7), malignant tumors (n = 16), and severely distorted or deformed brachial arteries that were difficult to detect (n = 2). Totally 389 patients were excluded based on the exclusion criteria, and another 365 patients were also not eligible due to lack of flow-mediated dilation (FMD) data. As a result, 1752 EH patients were finally included in this study. According to the time sequence, EH patients enrolled from August 2000 to December 2013 were the training group (n = 1204), and those enrolled from January 2014 to May 2016 were the validation group (n = 548). The training group was further divided into the CAD group (n = 263) and the non-CAD group (n = 941) according to whether the patients developed CAD or not (Fig. 1).

### Basic clinical data

Basic clinical data of EH patients were retrospectively obtained from the database of the research: Target organ damage and related risk factors in hypertensives, a clinical study conducted in Fuzhou city of China [16]. Specifically, the following data were recorded: (1) Demographic information: age, sex, course of EH, past medical history, family history of cardiovascular disease, place of residence (urban and rural area); (2) Lifestyle behavior: smoking; (3) Physical examination indexes: blood pressure and waist circumference; (4) Biochemical indicators: elbow venous blood was drawn in the morning after 8 h fasting, and blood lipid, liver function, kidney function, uric acid and white blood cells were detected by HITACH7170A biochemical instrument.

The diagnosis of CAD was based on *Guideline on the Diagnosis and Treatment of Stable Coronary Artery Disease (2018 Edition)* [17], while that of diabetes mellitus was based on *Guidelines for the Prevention and Control of Type 2 Diabetes in China (2017 Edition)* [18]. Smoking in this study was defined as ≥ 1 cigarette/day, lasting ≥ 6 months and still in progress [19], and abdominal obesity was defined as male waist circumference of ≥ 90 cm or female waist circumference of ≥ 85 cm [19].

### Determination of vasodilatory function

A non-invasive and reliable ultrasound method was used to detect vascular endothelial function by referring to the foreign operating guidelines [20] and the previous studies [12, 16, 21–24]. FMD was measured by LOGIQ7 color Doppler ultrasound (GE, America) in Fujian Hypertension Research Institute. Specifically, with the patients in the supine position, 5 cm of brachial artery above the right elbow line was the target vessel and marked with lines. After 10 min rest, the inner diameter of the brachial artery was measured three times in the resting state (the mean value was D_0_), and then the cuff was automatically inflated at 5–17.5 cm above the elbow joint to “200 mmHg or systolic blood pressure + 50mmHg”. The blood flow was completely blocked for 5 min, and then the inner diameter of the target vessel was measured after decompression for 80–90 s (D_1_) [12, 16, 21–24]. FMD was calculated according to the formula FMD = (D_1_ – D_0_)∗100%/D_0_, and endothelial dysfunction was defined as FMD of brachial artery ≤ 7.1% [6].

The three researchers who measured vasodilatory function all received strict and standardized training and used the same measurement method. In addition, 10 subjects (male and female half) were included for quality control measurement under the same condition. The coefficient of variation of the operators themselves was 1.9%, while the coefficient of variation among different operators was 6.92% [12,16].

### Statistical analysis

The normality of continuous variables was tested by Shapiro Wilk method; data conforming to normal distribution were expressed as mean ± standard deviation (SD), and data with non-normal distribution were expressed as median [25th, 75th percentile]. Counting data were presented as absolute values and percentages.

First, this study compared the baseline characteristics between the training group and the validation group. Secondly, independent sample t-test, Mann Whitney U-test and Person chi square test were selected to compare the variables between the CAD group and non-CAD group. Thirdly, the above differential variables obtained in the second step were subjected to the univariate logistic regression to screen out potential predictors of CAD. Potential predictors with *P* < 0.05 were included in multivariate logistic regression and modeled using the backward stepwise method. Finally, a nomogram was constructed after proper dichotomy of the above screened continuous variables. The predictive power of the nomogram was measured by calculating the area under curve (AUC) of receiver operating characteristic (ROC) analysis. The model was verified by using bootstrap self-sampling 1000 times. The calibration was judged by the mean absolute error (MAE) of the calibration curve and by Hosmer-Lemeshow test. The decision curve was conducted to evaluate the clinical applicability of the model. The difference was statistically significant with *P* < 0.05. SPSS 17.0 software (SPSS Inc. Chicago, America) and R software (version 4.2.1; https://www.R-project.org) were used for data analysis.

## Results

### Comparison of baseline characteristics between the training and validation groups

A total of 1752 patients were included in this study, with 1204 (68.7%) in the training group and 548 (31.3%) in the validation group. Compared with the validation group, the aspartate aminotransferase (AST), creatinine and total cholesterol in the training group were higher, and the proportion of EH treatment was relatively lower (*P* < 0.05). However, the values of these indicators (AST, creatinine and total cholesterol) were within the normal range. The two groups showed no statistical difference in other demographic characteristics, clinical indicators and FMD (*P* > 0.05). Collectively, the baseline characteristics of the training and validation groups were comparable (Table 1).

**Table 1:** Baseline characteristics between the training group and the validation group

Variables	Training group (n = 1204)	Validation group (n = 548)	*P* value
With CAD	263 (21.8%)	76 (13.9%)	
Age, years^a^	61.7 ± 12.5	60.6 ± 13.4	0.120
Male^b^	695 (57.7%)	295 (53.8%)	0.128
Waist circumference, cm^a^	87.9 ± 9.4	87.2 ± 8.4	0.119
Duration of EH, years^c^	2.0 [1.0,10.0]	4.0 [0.0,10.0]	0.563
Smoking^b^	166 (13.8%)	61 (11.1%)	0.125
Family history of cardiovascular disease^b^	219 (18.2%)	103 (18.8%)	0.761
EH treatment^b^	508(42.2%)	272 (49.6%)	0.004*
Urban^b^	505 (41.9%)	252 (46.0%)	0.113
Systolic blood pressure, mmHg^a^	135.8 ± 15.0	134.7 ± 17.9	0.212
Diastolic blood pressure, mmHg^a^	79.9 ± 11.0	80.8 ± 11.1	0.105
Diabetes mellitus^b^	152 (12.6%)	63 (11.5%)	0.505
Biochemical indicators			
White blood cells, 10^9^/L^a^	6.0 ± 1.7	6.0 ± 1.5	0.891
Total bilirubin, umol/L^a^	13.9 ± 5.4	13.8 ± 5.4	0.696
AST, U/L^a^	26.5 ± 9.5	25.1 ± 7.4	0.001*
Creatinine, umol/L^a^	72.9 ± 19.0	66.1 ± 16.2	< 0.001*
Glucose, mmol/L^a^	5.8 ± 1.3	5.8 ± 1.3	0.464
Uric acid, mmol/L^a^	348.7 ± 94.7	353.8 ± 87.8	0.362
Total cholesterol, mmol/L^a^	5.0 ± 1.1	4.8 ± 1.2	< 0.001*
Triglyceride, mmol/L^c^	2.0 [1.0,10.0]	1.3 [0.9,1.8]	0.068
LDL-C, mmol/L^a^	3.0 ± 1.0	3.0 ± 1.0	0.358
HDL-C, mmol/L^a^	1.4 ± 0.4	1.4 ± 0.5	0.079
FMD (%)^a^	9.4 ± 5.8	9.9 ± 5.5	0.088

EH, essential hypertension; CAD, coronary artery disease; AST, aspartate aminotransferase; LDL-C, low density lipoprotein cholesterol; HDL-C, high density lipoprotein cholesterol; FMD, flow-mediated dilation

*P < 0.05, ^a^compared by independent sample t-test, ^b^compared by Person chi square test, ^c^compared by Mann Whitney U-test

### Risk factors for CAD in EH patients in the training group

The 1204 EH patients in the training group were divided into the CAD group (n = 263, 21.8%) and non-CAD group (941, 78.2%). Univariate analysis demonstrated that FMD and traditional risk factors (age, duration of EH, smoking, waist circumference and diabetes mellitus) were all related to EH complicated with CAD (*P* < 0.05), as shown in Table 2. Then, all the six variables passed the univariate logistic regression test and were all included in multivariate logistic regression analysis. Ultimately, the results showed that FMD, age, duration of EH, waist circumference, and diabetes mellitus were independently associated with CAD in EH patients (*P* < 0.05). Although smoking did not reach statistical significance (*P* = 0.062), it was still included in the regression model fitting effect (Table 3).

**Table 2:** Comparison of clinical characteristics of hypertensives with and without CAD in the training group

Variables	Non-CAD group (n = 941)	CAD group (n = 263)	*P* value
China-PAR			
Age, years^a^	60.1 ± 12.5	67.2 ± 10.9	< 0.001*
Male^b^	530 (56.3%)	165 (62.7%)	0.063
Waist circumference, cm^a^	87.3 ± 9.3	90.2 ± 9.2	< 0.001*
Smoking^b^	92 (9.78%)	74 (28.1%)	< 0.001*
Family history of cardiovascular disease^b^	180 (19.1%)	39 (14.8%)	0.110
EH treatment^b^	600 (63.8%)	180 (68.4%)	0.160
Northerner	0(0%)	0(0%)	
Urban^b^	385 (40.9%)	120 (45.6%)	0.171
Systolic blood pressure, mmHg^a^	135.5 ± 15.1	137.2 ± 14.9	0.098
Diabetes mellitus^b^	90 (9.6%)	62 (23.6%)	< 0.001*
Total cholesterol, mmol/L^a^	5.0 ± 1.1	4.9 ± 1.1	0.288
HDL-C, mmol/L^a^	1.4 ± 0.4	1.4 ± 0.4	0.080
Duration of EH disease, years^c^	1.5 [0.5,7.0]	6.0 [1.0,10.0]	< 0.001*
FMD (%)^a^	10.4 ± 5.8	5.6 ± 4.3	< 0.001*

EH, essential hypertension; CAD, coronary artery disease; HDL-C, high density lipoprotein cholesterol; FMD, flow-mediated dilation

*P < 0.05, ^a^compared by independent sample t-test, ^b^compared by Person chi square test, ^c^compared by Mann Whitney U-test

**Table 3:** Univariate and multivariable logistic regression analysis of six variables in the training group

Variables	Univariate				Multivariate		
	*OR*	95%*CI*	*P* value		*OR*	95%*CI*	*P* value
Age	1.053	1.040–1.067	< 0.001*		1.046	1.030–1.061	< 0.001*
Waist circumference	1.033	1.018–1.049	< 0.001*		1.018	1.001–1.035	0.043*
Smoking	3.613	2.561–5.098	< 0.001*		1.471	0.980–2.206	0.062
Diabetes mellitus	2.917	2.039–4.173	< 0.001*		2.308	1.524–3.496	< 0.001*
Duration of EH disease	1.050	1.034–1.067	< 0.001*		1.021	1.002–1.040	0.027*
FMD	0.807	0.777–0.837	< 0.001*		0.817	0.785–0.850	< 0.001*

EH, essential hypertension; FMD, flow-mediated dilation; OR, odds ratio; CI, confidence interval. *P < 0.05

### Development of a nomogram prediction model

**Fig. 2:**
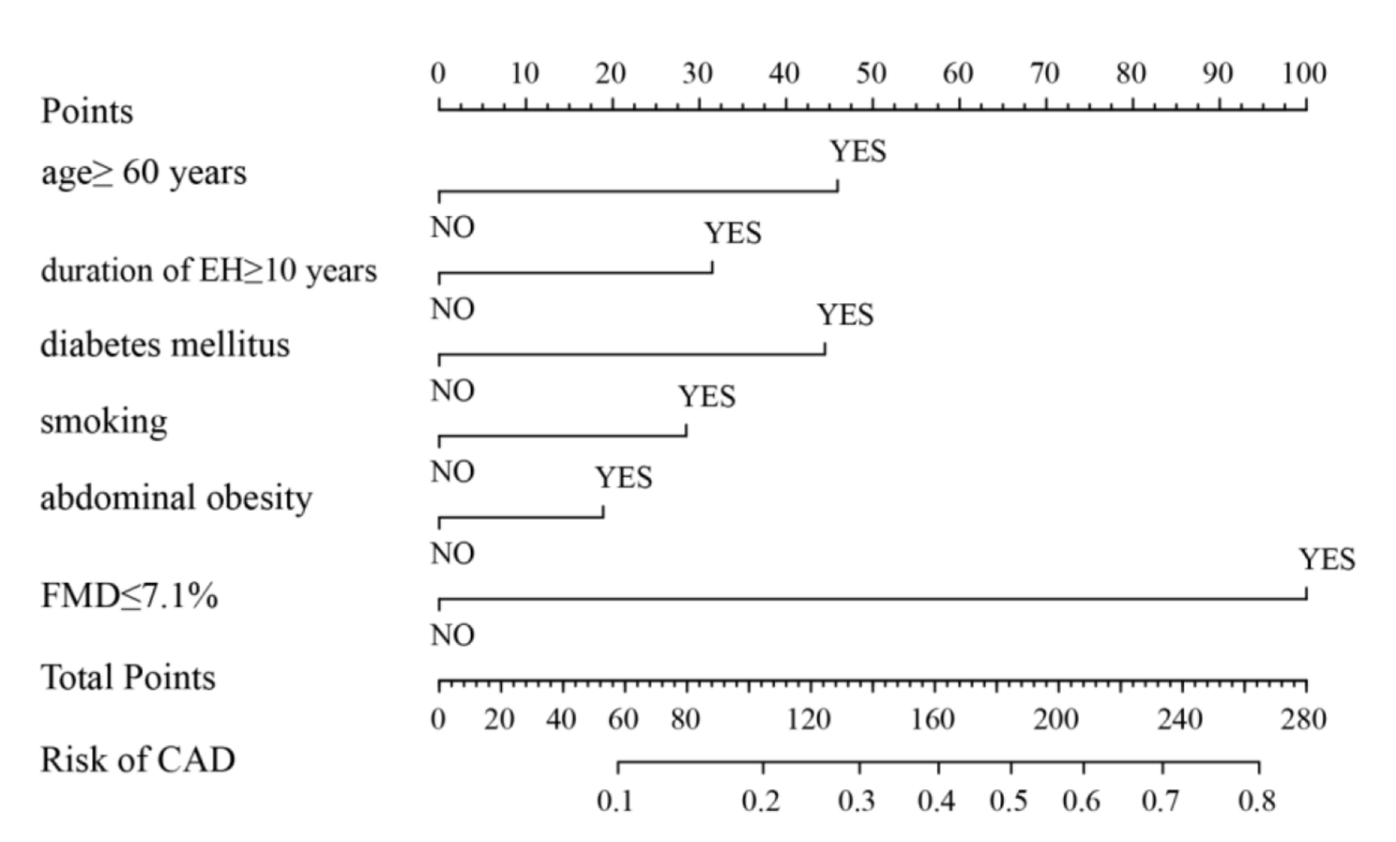
The CAD risk nomogram was developed using the predictors, including FMD and traditional risk factors. CAD, coronary artery disease; FMD, flow-mediated dilation

In binary assignment of continuous variables, FMD ≤ 7.1% was recorded as 1, otherwise as 0; over 60-year-old was recorded as 1, otherwise as 0; the duration of EH ≥ 10 years was denoted as 1, otherwise as 0; abdominal obesity was recorded as 1, otherwise as 0. Then, all the six binary variables were used as the predictors to build the nomogram model for predicting CAD in EH patients (Fig. 2). The nomogram was interpreted as follows. First, vertical lines were made on the horizontal axis of each predictor of a patient to derive the corresponding score. Then the individual points were then summed to obtain the total point. Finally, the risk of CAD in each EH patient could be calculated based on the total point.

### Validation and evaluation of the nomogram

**Fig. 3:**
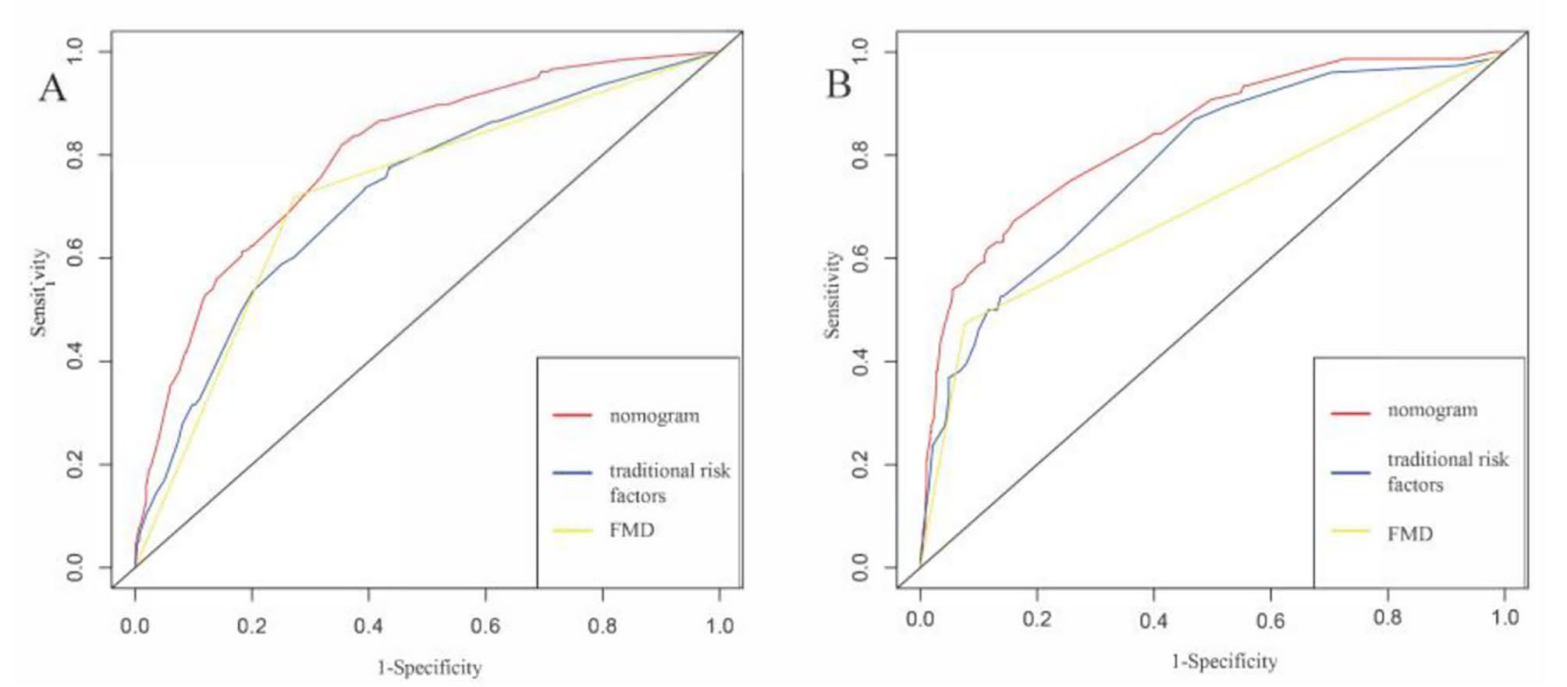
ROC curves for predicting essential hypertension complicated with coronary artery disease in training group **(A)** and validation group **(B)**

**Fig. 4:**
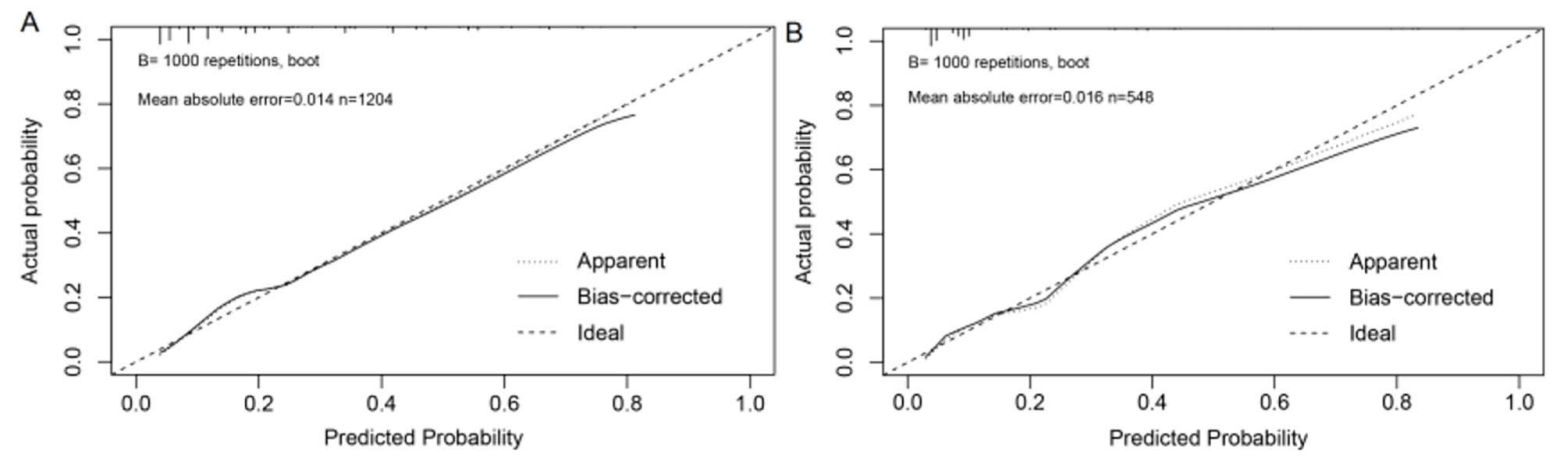
Calibration curves of training **(A)** and validation **(B)** groups. The diagonal dotted line represents the ideal curve, the solid line represents the bias-correction curve, and the dashed line represents the apparent curve

**Fig. 5:**
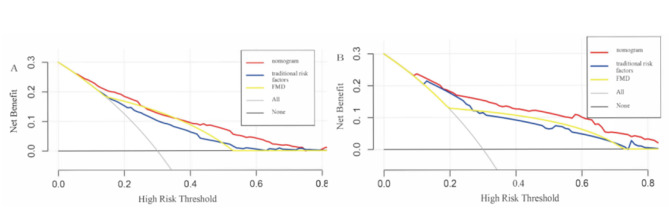
Decision curve analyses of training **(A)** and validation groups **(B)**

First of all, the ROC curves of the nomogram were drawn for the training group and the validation group, respectively. AUC of the training group was 0.799 (95%*CI* 0.770–0.829), and that of the validation group was 0.836 (95%*CI* 0.787–0.886). AUC of the nomogram model in both groups were larger than the AUC of the “traditional risk factors” and “FMD” models, indicating that the nomogram model had good prediction ability (Fig. 3). Then, the Hosmer-Lemeshow test was conducted in the training group and the validation group respectively, and the calibration curve (Fig. 4) was drawn to evaluate the model calibration ability. The results showed that the predictive model had good calibration in both groups (training group: *χ*^*2*^ = 0.55, *P* = 0.759; validation group: *χ*^*2*^ = 1.62, *P* = 0.446; *P* > 0.05 indicated that the predictive model worked well.). Finally, decision curves were also drawn in both groups respectively, and the results showed that compared with the “traditional risk factors” and “FMD” models, the nomogram had better clinical application value in predicting CAD in EH patients (Fig. 5).

## Discussion

In the present study, FMD, age, duration of EH, smoking, waist circumference, and diabetes mellitus were predictors of CAD in EH patients. Using these predictors, a nomogram was constructed. AUC values of the model were 0.799 in the training group and 0.836 in the validation group, respectively, indicating that the nomogram had good prediction ability. Furthermore, Hosmer-Lemeshow test and decision curve analysis were performed and found good predictive stability and clinical application value of the nomogram in predicting CAD in HE patients.

This study showed that the prevalence rate of EH combined with CAD was 19.3%, which was similar to the 21.3% reported by Zhang et al [25]. Given the high prevalence rate, it is necessary to actively carry out CAD screening and prevention for EH patients to reduce ASCVD events. Previous researches have suggested that endothelial dysfunction is closely related to ASCVD and can be an important pathophysiological feature of ASCVD [8, 9]. Endothelial function indexes have independent predictive value in assessing the incidence of CAD [6, 7]. FMD is a mature, accurate and non-invasive indicator of endothelial function of peripheral artery [16, 21–24]. Although FMD is not a “gold standard” for diagnosing CAD, it is significantly related to endothelial function of coronary artery [8], and contributes to clinical prediction of CAD [9]. In our study, the analysis of 1752 EH patients found that FMD was an independent predictive factor for CAD in EH patients; compared with EH patients with FMD > 7.1%, FMD ≤ 7.1% could increase the weight of 100 points in the nomogram and contributed the most to CAD risk. Wang et al. [26] also found similar results through the study of 151 diabetic patients; FMD was independently associated with CAD, and could predict the degree of coronary artery stenosis alone or in combination with ankle brachial index. At present, the cut-off value of endothelial dysfunction defined by FMD is controversial. ≤ 7% and 7.1% were defined as endothelial dysfunction in two prospective studies in Japan, respectively [6, 27], while in western countries, a lower cut-off point of 4-5% was taken [28, 29]. Because of the enrolled patients were Asian, 7.1% was selected as the cut-off point for analysis. Our results suggest that EH patients with endothelial dysfunction have a higher risk of CAD. Therefore, for EH patients with FMD ≤ 7.1%, early screening for CAD risk and protection of endothelial function are needed to reduce the incidence of ASCVD events.

In addition to FMD, the traditional risk factors including age, duration of EH, smoking, waist circumference and diabetes mellitus were also predictive factors of CAD in EH patients, which was consistent with previous research results [5]. CAD is a kind of senile cardiovascular disease, with a prevalence of 10.2% in people aged ≥ 15 years but 27.8% in people aged ≥ 60 years [30]. At the same time, with the increase of EH duration and the continuous progress of atherosclerosis, the incidence of CAD will also increase [31]. This study found that the risk of combined CAD respectively increased by 46% and 21% for each 10-year increase in age and EH duration in EH patients. Therefore, elderly EH patients with a long course of disease need more active evaluation of CAD, early diagnosis, and early treatment. Studies have confirmed that smoking is one of the traditional risk factors of CAD [5, 13, 14], and its mechanism may be related to oxidative stress and inflammation [32]. This study found that smokers with EH were 1.47 times more likely to develop CAD than non-smokers, suggesting that quitting smoking is of great significance for the prevention and treatment of CAD. Abdominal obesity and diabetes mellitus are related to metabolic diseases [18], and also act as important risk factors for ASCVD [26]. Our result demonstrated that for EH patients, CAD risk increased by nearly 20% for every 10 cm increase in waist circumference, and EH patients were 2.31 times more likely to develop CAD than those without diabetes mellitus. Therefore, it is necessary to make the majority of EH patients understand the harm of abdominal obesity through health education, and motivate them to actively improve their lifestyle, control their weight, and monitor blood glucose.

The prediction model in this study is established by combining FMD and traditional risk factors. The traditional risk factors are acquired from China-PAR study [5]. According to the actual situation in China, the study also included risk factors with “Chinese characteristics” on the basis of traditional ASCVD risk factors in Europe and America, which is more suitable for Chinese people. Different from existing models, our prediction model included FMD as a risk factor to emphasize the relationship between endothelial function and CAD in EH population. Due to the limitations of traditional risk factor prediction, it is difficult to make further breakthroughs in the discrimination of CAD prediction models at home and abroad at present [5, 13, 14]. The innovation of our study is the inclusion of FMD, an indicator of endothelial function, in the prediction model for the first time, and the model shows good accuracy. In conclusion, the nomogram constructed in this study has a good prediction for combined CAD in EH patients, which is generally equivalent to China-PAR model [5] and PCE model [14], but the predictive factors of the nomogram are more concise, intuitive, and suitable for clinical application. The calibration curve and Hosmer-Lemeshow test confirmed that the predicted results of the model were in good agreement with the actual results, and the decision curve showed that the model could bring clinical benefits to patients.

There are some certain limitations in this study. Firstly, it was a retrospective study that was impossible to determine the “causal relationship” of the disease. Secondly, it was a single-center study with all cases from southern China and a large time span of inclusion of cases. Therefore, there was inevitably case selection bias. Finally, the sample size of the model was relatively small, lacking external validation of data from other centers, so the model needs to be further verified by external large sample data in clinical promotion and application.

## Conclusion

To sum up, this study showed that FMD and traditional risk factors (including age, duration of EH, smoking, waist circumference and diabetes mellitus) were independent predictors for CAD in EH patients. The practical nomogram from the above elements might identify the high-risk groups of CAD and provide early individualized prevention strategies for EH.

## Data Availability

The data used to support the findings of this study are available from the corresponding author upon request.
